# Early detection of unhealthy behaviors, the prevalence and receipt of antiviral treatment for disabled adult hepatitis B and C carriers

**DOI:** 10.1186/s12889-016-2844-0

**Published:** 2016-02-13

**Authors:** Sui-Whi Jane, Ming-Shyan Lin, Wen-Nan Chiu, Li-Ju Lai, Po-Han Chen, Mei-Yen Chen

**Affiliations:** Nursing Department, Chang Gung University of Science and Technology, Taoyuan, Taiwan; Deparment of Internal Medicine, Chang Gung Memorial Hospital, Yunlin, Taiwan; Department of Ophthalmology, Chang Gung Memorial Hospital, Chiayi, Taiwan; Department of Orthopedic Surgery, Chang Gung Memorial Hospital, Yunlin, Taiwan; College of Nursing, Chang Gung University of Science and Technology, No. 2, Chiapu Rd. West Sec., Putz City, Chiayi County 61363 Taiwan

**Keywords:** Prevalence, Unhealthy behavior, Hepatitis B virus, Hepatitis C virus, Disability

## Abstract

**Background:**

Evidence indicates that hepatitis B virus (HBV) and hepatitis C virus (HCV) infections are the leading causes of liver cirrhosis and hepatocellular carcinoma. Antiviral treatments have recently been reported as successful cures. However, the prevalence rates of HBV or HCV infection, unhealthy behaviors and receipt of adequate treatment in disabled adults have not been described. The aim of this study was to examine the prevalence of HBV or HCV carriers, receipt of antiviral treatment, and early detection of unhealthy behaviors in disabled adults in Taiwan.

**Methods:**

A population-based, cross-sectional study was conducted between July and December 2013 with 845 community-dwelling adults with disabilities aged >20 years. Statistical analyses included descriptive statistics, Chi-squared tests, and stepwise regression analysis.

**Results:**

The prevalence of HBV and HCV infections was 12.9 and 14.1 %, respectively. HCV carriers tended to be older (*p* < 0.001) and with a lower education (*p* < 0.001). The majority of HBV/HCV carriers did not know the type of hepatitis infection and did not receive adequate antiviral treatment. After adjusting for potential confounding variables, regression analysis showed that the factors significantly associated with elevated liver function were HCV infection (*p* < 0.001), HBV infection (*p* = 0.001), high fasting blood glucose levels (*p* = 0.001), overweight (*p* = 0.003), older age (*p* = 0.027), and alcohol drinking (*p* = 0.028).

**Conclusions:**

There was a high prevalence of HCV infection among adults with disabilities; few received adequate antiviral treatment or early detection of unhealthy behaviors for the prevention of liver cancer. Clinicians can provide health education to help the participants and caregivers better understand the relationships between specific risk factors and liver health and can encourage HBV and HCV carriers to undergo annual physical check-ups and receive adequate treatment, as covered by the national health insurance.

## Background

Liver cancer is one of the most common cancers worldwide, particularly in Central and East Asia [1]. The leading causes of liver cirrhosis, liver failure, and hepatocellular carcinoma (HCC) are hepatitis B virus (HBV) and hepatitis C virus (HCV) infections. Hepatitis is inflammation of the liver, most commonly caused by a viral infection [1–4]. The mechanism by which hepatitis viral infection leads to liver cancer might involve the infection-related chronic inflammation [3, 4].

Globally, HBV and HCV infections and their sequelae remain a major health problem, associated with 1.45 million deaths annually [5]. In Taiwan, liver cirrhosis and HCC are the most common causes of death, accounting for 34.9 deaths per 100,000 persons [6]. In addition, HBV and HCV are endemic, with prevalence rates of 15–20 and 2–3 %, respectively, among the general adult population. An estimated 2.5–3.0 million people are HBV carriers, and nearly 90 % of HCC patients are infected with HBV or HCV. Furthermore, 60–90 % of carriers are at risk of liver cirrhosis and HCC [6, 7]. In Taiwan, HCV infection is significantly associated with health disparity and older people in disadvantaged areas [8–10].

HBV infection occurs mainly during early childhood, and mother-to-infant transmission accounts for the majority of cases with chronic infection [3]. The majority of HCV infections are transmitted via contact with the blood of an infected person or with unsterilized materials, such as injectable drug equipment, razors, or tattoo needles [4]. Fortunately, Taiwan launched a universal HBV vaccination program in 1984, in which three HBV vaccine doses were administered to all infants starting from the first week of life [3]. Long-term studies have demonstrated that 20 years after the mass HBV vaccination program was implemented, the incidence of HBV infection significantly declined to 0.6 % in young adults [11, 12]. Additionally, antiviral treatments for HBV and HCV in adult carriers, such as interferon combined with ribavirin, have recently been reported as successful cures [1–4]. However, many people still receive inadequate treatment, especially disabled residents living in rural areas [10].

In Taiwan, more than one million people were designated as disabled by the government in 2012 [13]. The top five disabilities were physical disability, loss of vital organs, hearing impairment, intellectual disability, and a combination of disabilities. People with disabilities have poorer health outcomes, less education, less economic participation, and higher rates of poverty than people without disabilities [14]. Therefore, disability has become a human rights issue in Taiwan; the potential health care savings due to early intervention and prevention efforts have also recently been recognized [13].

Liver dysfunction, such as abnormal serum glutamate-pyruvate transaminase (SGPT) levels, and HCC have been associated not only with HBV or HCV infection but also with cigarette smoking, alcohol drinking, and obesity [10, 15–18]. In addition, obesity is an increasingly important health problem in disabled people [19]. Therefore, reductions in weight might reverse the burden of liver function; strategies for weight loss include adopting a healthier diet (e.g., 3 servings of vegetables and 2 servings of fruit daily), drinking plenty of water (e.g., 1500 mL/day), and exercising (e.g., 30 min/day, 3 times per week) [20].

Few actions have been implemented to enhance cancer prevention policies among HBV or HCV carriers in disadvantaged areas. Moreover, few studies have explored the prevalence of and relationships with unhealthy behaviors, health status, and receipt of adequate treatment among disabled HBV or HCV carriers. In this study, we investigated the prevalence of and relationships between unhealthy behaviors and determinants of liver function among disabled HBV/HCV carriers.

## Methods

### Participants and design

This study was part of a health promotion program for community-dwelling adults with disabilities led by a nursing team in collaboration with a private hospital and the Bureau of Health Promotion in Chiayi County, Taiwan. This cross-sectional, descriptive study aimed to explore the prevalence of HBV/HCV carriers and receipt of anti-viral treatment in the southwestern coastal area. In this area, there were 37,629 people with disabilities, based on government records [13]; from these records, participants were selected by convenience sampling. The inclusion criteria were the ability to (1) complete the questionnaire in writing or by interview in Mandarin or Taiwanese with or without assistance or via a caregiver, (2) walk or transfer to the local hospital with or without assistance from family or institutional staff, and (3) provide written informed consent before study enrollment. Participants who were unable to answer questions, who had severe chronic disease or cancer, or who were undergoing dialysis were excluded.

A community-based health screening survey was conducted between July and December 2013, and 1024 disabled residents participated in this project. From this information, we determined the prevalence rates of viral infections, receipt of adequate antiviral treatments, unhealthy behaviors, and risk factors associated with liver function among disabled HBV/HCV carriers.

### Instruments and measurements

A. *Demographic characteristics* included age, gender, education level (years of education achieved), marital status, occupation, living arrangement, and disability classification. Disabilities were classified according to the government definition (e.g., physical, intellectual, and multiple disabilities) and confirmed before the interview was conducted.
B. *Unhealthy behaviors and receipt of anti-virus treatment* were assessed based on expert recommendations related to adopting healthy lifestyles and habits for the liver [10, 20] using eight questions (1) “Do you regularly drink alcohol?” Drinking was classified as “never” if the participant had never consumed alcohol, “ceased” if the participant had previously consumed alcohol but had ceased drinking alcohol for at least 6 months, or “current” if the participant was currently drinking. (2) “Do you smoke cigarettes?” Smoking was classified as “never” if the participant never smoked, “ceased” if the participant had previously smoked but had ceased smoking for at least 6 months, or “current” if the participant was currently smoking. (3) Regular exercise was classified as “irregular” if the participant responded never or sometimes or as “regular” if the participant usually exercised for a total or cumulative duration of >30 min per day, three times per week, or 150 min per week. (4) “How often do you consume at least 1500 mL or eight bowl-sized cups of water each day?” The answer was classified as “insufficient” if the participant answered never or sometimes and as “sufficient” if the participant answered usually or always. (5) “Do you eat 3 servings of vegetables every day?” The answer was classified as “insufficient” if the participant answered never or sometimes and as “sufficient” if the participant usually consumed at least 3 servings or 1.5 bowl-sized servings of vegetables per day. (6) “Do you eat 2 servings of fruit every day?” The answer was classified as “insufficient” if the participant answered never or sometimes and as “sufficient” if the participant usually consumed at least 2 servings or one bowl-sized serving of fruit per day. (7) “Do you know that you have a viral infection called hepatitis?” If yes, then the participant was asked (8) “Have you received hepatitis treatment, such as an anti-viral prescription from a physician?”
C. *Physiological biomarkers* were measured for liver function, including HBV, HCV, and SGPT (normal range, <40 mU/mL) as measured by the collaborating hospital during the physical examination and serum hepatitis B surface antigen (HBsAg) and anti-HCV antibodies as determined using enzyme-linked immunoassays; fasting blood glucose (normal range, <110 mg/dL); systolic and diastolic blood pressure (normal range, <140/90 mmHg); and body mass index (BMI), as calculated by weight (kg) divided by the square of height (m^2^). All standard references were from the Ministry of Health and Welfare [21].

### Procedure and ethical considerations

This study was approved by the Ethical Committee of the Institutional Review Board (Chang Gung Memorial Hospital No 102-3331B). Written informed consent was obtained from all participants or their caregiver, after describing the study purpose and explaining that a free medical examination would be provided. A cover letter inviting participation in the study was sent to the 18 district public health nurses; this letter emphasized that the responses would remain confidential. Participants were allowed to ask questions and seek clarification about the study before they consented to participate. All participants were interviewed during a weekend in a school auditorium after the blood collection and physical examination by the staff of the collaborating hospital. To help build a caring environment, each participant was accompanied by a senior nursing student and community volunteer during the health screenings.

### Data analysis

SPSS version 20 (IBM Corp., Armonk, NY) was used for data analyses. All tests were two-sided, and *p* values <0.05 were considered statistically significant. The Chi-square statistic for testing the equality of proportions or rates was used to compare demographic variables, physiological biomarkers, and unhealthy behaviors between the individuals with or without HBV or HCV infection. Multivariate linear regression was used to analyze the factors associated with liver function that were chosen on the basis of relevant confounders after univariate analysis.

## Results

### Demographic characteristics

Of the 1024 disabled community residents who were eligible, 177 did not meet the inclusion criteria, resulting in 847 participants for data analyses. After excluding 1 participant who failed to complete the physical examination and 1 participant who had an incomplete questionnaire, the final data analyses were conducted with 845 participants. The mean age was 53.9 years (SD = 18.4; range 20–96), and the majority of the participants were men (*n* = 472, 55.8 %). Nearly one-third of the participants (*n* = 276, 32.6 %) had a physical disability, 27 % (*n* = 227) had an intellectual disability, and the remainder (*n* = 342, 40.4 %) had multiple disabilities. Nearly half (50.8 %) did not have an education beyond middle school. More than two-thirds of the participants were unemployed (*n* = 609), 49 % were married, and more than two-thirds (78.9 %) were living with their family, while the remainder lived with health care assistants or alone (Table 1).

**Table 1:** Demographic characteristics of the samples (*N* = 845)

Variables	Hepatitis B infection		Hepatitis C infection	
	Negative	Positive	Negative	Positive
	N (%)		N (%)	
Gender	*χ* ^2^ = 2.16		*χ* ^2^ = 0.01	
Female	332 (89.0)	41 (11.0)	320 (85.8)	53 (14.2)
Male	404 (85.5)	68 (14.4)	406 (86.0)	66 (14.0)
Age (years) Mean ± SD	51.5 ± 14.7	*χ* ^2^ = 11.76**	62.5 ± 15.7	*χ* ^2^ = 24.63***
~ 39	184 (87.2)	27 (12.8)	198 (93.8)	13 (6.2)
40 ~ 64	307 (83.2)	62 (16.8)	321 (87.0)	48 (13.0)
≥ 65	245 (92.5)	20 (7.5)	207 (78.1)	58 (21.9)^a^
Disability classification	*χ* ^2^ = 1.21		*χ* ^2^ = 7.16*	
Physical	243 (88.0)	33 (12.0)	231 (83.7)	45 (16.3)
Intellectual	193 (85.0)	34 (15.0)	207 (91.2)	20 (8.8)
Multiple disabilities	300 (87.7)	42 (12.3)	288 (84.2)	54 (15.8)
Education	*χ* ^2^ = 0.84		*χ* ^2^ = 16.36***	
≤ Primary school	378 (88.1)	51 (11.9)	348 (81.1)	81 (18.9)
≥ Middle school	356 (86.0)	58 (14.0)	376 (90.8)	38 (9.2)
Occupation	*χ* ^2^ = 0.11		*χ* ^2^ = 5.09*	
With job	207 (87.7)	29 (12.3)	213 (90.3)	23 (9.7)
Without job	529 (86.9)	80 (13.1)	513 (84.2)	96 (15.8)
Marital status^a^	*χ* ^2^ = 6.48*		*χ* ^2^ = 13.56***	
Single	269 (85.4)	46 (14.6)	288 (91.4)	27 (8.6)
Married	371 (89.8)	42 (10.2)	344 (83.3)	69 (16.7)
Others	94 (81.7)	21 (18.3)	92 (80.0)	23 (20.0)
Living arrangement	*χ* ^2^ = 0.06		*χ* ^2^ = 0.22	
With families	580 (87.0)	87 (13.0)	575 (86.2)	92 (13.8)
Other	156 (87.6)	22 (12.4)	151 (84.8)	27 (15.2)

Pearson Chi-Square: ^*^ *p* < 0.05; ^**^ *p* < 0.01; ***p < 0.001

^a^with missing data

The prevalence of HBV and HCV infections was 12.9 % (*n* = 109) and 14.1 % (*n* = 119), respectively (Table 1). Based on HBV infection, there were no statistically significant differences in gender, disability classification, education level, occupation status, and living arrangement; however, age and marital status were significantly different. Based on HCV infection, there were no significant differences in gender and living arrangement; however, the HCV carriers tended to be older (*χ*^2^ = 24.6, *p* < 0.001) and have a lower level of education (*χ*^2^ = 16.4, *p* < 0.001) than the non-carriers.

### Unhealthy behaviors between hepatitis B or C virus carriers

Of the total sample, 77 (9.1 %) and 121 (14.3 %) participants were current users of alcohol and cigarette smokers, respectively. More than half (53.5 %, *n* = 452) reported irregular exercise, 66.7 % (*n* = 563) reported insufficient water intake, 81 % (*n* = 685) reported insufficient fruit intake, and 67.5 % (*n* = 570) reported insufficient vegetable intake. There were no significant differences between HBV/HCV carriers and non-carriers in any of the health-related behaviors (Table 2). Regarding knowledge of hepatitis viral infection, the majority of HBV/HCV carriers did not know the type of hepatitis infection and had not received adequate anti-viral treatment (*p* < 0.001, Table 2).

**Table 2:** Health-related behaviors among hepatitis B or C virus carriers

Variables	Hepatitis B infection		Hepatitis C infection	
	Negative	Positive	Negative	Positive
	N (%)		N (%)	
Alcohol use^a^	*χ* ^2^ = 4.88		*χ* ^2^ = 0.41	
Never	625 (85.1)	85 (78.0)	608 (84.0)	102 (85.7)
Current user	61 (8.3)	16 (14.7)	68 (9.4)	9 (7.6)
Cessation	48 (6.5)	8 (7.3)	48 (6.6)	8 (6.7)
Cigarette smoke	*χ* ^2^ = 3.56		*χ* ^2^ = 0.43	
Never	555 (75.4)	75 (68.8)	544 (74.9)	86 (72.3)
Current smokers	99 (13.5)	22 (20.2)	103 (14.2)	18 (15.1)
Cessation	82 (11.1)	12 (11.0)	79 (10.9)	15 (12.6)
Adopting regular exercise^a^	*χ* ^2^ = 1.86		*χ* ^2^ = 0.40	
Irregular	387 (52.7)	65 (59.6)	392 (54.0)	60 (50.8)
Regular	348 (47.3)	44 (40.4)	334 (46.0)	58 (49.2)
Water (1500 mL/day)^a^	*χ* ^2^ = 0.21		*χ* ^2^ = 0.26	
Sufficient	245 (33.4)	34 (31.2)	242 (33.5)	37 (31.1)
Insufficient	488 (66.6)	75 (68.8)	481 (66.5)	82 (68.9)
Fruit (2 servings/day)	*χ* ^2^ = 0.01		*χ* ^2^ = 0.77	
Sufficient	139 (18.9)	21 (19.3)	134 (18.5)	26 (21.8)
Insufficient	597 (81.1)	88 (80.7)	592 (81.5)	93 (78.2)
Vegetable (3 servings/day)	*χ* ^2^ = 0.01		*χ* ^2^ = 0.02	
Sufficient	239 (32.5)	36 (33.0)	237 (32.6)	38 (31.9)
Insufficient	497 (67.5)	73 (67.0)	489 (67.4)	81 (68.1)
Awareness of viral infection	*χ* ^2^ = 44.7***		*χ* ^2^ = 49.97***	
Yes	26 (3.5)	21 (19.3)	24 (3.3)	23 (19.3)
No	710 (96.5)	88 (80.7)	702 (96.7)	96 (80.7)
Receiving antiviral treatment	*χ* ^2^ = 21.31***		*χ* ^2^ = 30.56***	
Yes	12 (1.6)	10 (9.2)	10 (1.4)	12 (10.1)
No	724 (98.4)	99 (90.8)	716 (98.6)	107 (89.9)

***p < 0.001

^a^with missing data

### Physiological biomarkers and health status among hepatitis carriers

HCV carriers tended to have abnormal SGPT (*χ*^2^ = 53.72, *p* < 0.001) and systolic blood pressure (*χ*^2^ = 6.29, *p* < 0.05) levels (Table 3). Considering the health status of the HBV and HCV carriers, 49.5 and 58.7 % were overweight, respectively; 28.7 and 44.9 % had abnormal systolic blood pressure, respectively; and 18.3 and 26.9 % had high fasting blood glucose, respectively.

**Table 3:** Physiological biomarkers associated with hepatitis B or C virus carriers

Variables	Hepatitis B infection		Hepatitis C infection	
	Negative	Positive	Negative	Positive
	N (%)		N (%)	
SGPT (mu/mL)	*χ* ^2^ = 1.66		*χ* ^2^ = 53.72***	
< 40	635 (86.3)	89 (81.7)	648 (89.3)	76 (63.9)
≥ 40	101 (13.7)	20 (18.3)	78 (10.7)	43 (36.1)
Body mass index (kg/m^2^)	*χ* ^2^ = 1.87		*χ* ^2^ = 0.38	
Average/under	292 (43.2)	50 (50.5)	299 (44.6)	43 (41.3)
Overweight/Obesity	384 (56.8)	49 (49.5)	372 (55.4)	61 (58.7)
SBP (mmHg)	*χ* ^2^ = 1.98		*χ* ^2^ = 6.29*	
< 140	470 (64.4)	77 (71.3)	482 (66.9)	65 (55.1)
≥ 140	260 (35.6)	31 (28.7)	238 (33.1)	53 (44.9)
DBP (mmHg)	*χ* ^2^ = 0.54		*χ* ^2^ = 0.03	
< 90	558 (76.4)	86 (79.6)	554 (76.9)	90 (76.3)
≥ 90	172 (23.6)	22 (20.4)	166 (23.1)	28 (23.7)
FBG (mg/dL)	*χ* ^2^ = 0.66		*χ* ^2^ = 2.56	
< 109	575 (78.2)	89 (81.7)	577 (79.6)	87 (73.1)
≥ 110	160 (21.8)	20 (18.3)	148 (20.4)	32 (26.9)

*Abbreviations:* *SGPT* serum glutamic pyruvic transaminase, *SBP* systolic blood pressure, *DBP* diastolic blood pressure, *FBG* fasting blood glucose

^*^ *p* < 0.05; ***p < 0.001

After adjusting for potential confounding variables, HCV infection (β = 0.2, *p* < 0.001), HBV infection (β = 0.12, *p* = 0.001), high fasting blood glucose levels (β = 0.11, *p* = 0.001), overweight (β = 0.1, *p* = 0.003), older age (β = −0.08, *p* = 0.027), and current alcohol drinking (β = 0.08, *p* = 0.028) were significant in the regression analysis for elevated liver function (Table 4).

**Table 4:** Factors associated with elevated liver function (SGPT)

Variables	Unstandardized		Beta	t value	*p*	95 % CI^a^
	B	SE				
Constant	19.85	1.67		11.89	<0.001	16.58 ~ 23.13
HCV (1 = positive)	15.28	2.59	0.20	5.90	<0.001	10.19 ~ 20.37
HBV (1 = positive)	9.30	2.68	0.12	3.47	0.001	4.04 ~ 14.56
Fasting blood glucose (1 > 110 mg)	7.26	2.24	0.11	3.25	0.001	2.87 ~ 11.65
BMI (1 = overweight/obesity)	5.71	1.89	0.10	3.02	0.003	2.00 ~ 9.42
Age (1 > 65)	−4.40	1.98	−0.08	−2.22	0.027	−8.29 ~ −0.51
Alcohol (1 = current drinker)	6.15	2.78	0.08	2.21	0.028	0.68 ~ 11.61
Smoking (1 = current smoker)	1.91	2.35	0.03	0.81	0.418	−2.71 ~ 6.53

*Abbreviations*: *SGPT* serum glutamic pyruvic transaminase, *HCV* hepatitis C virus, *BMI* body mass index, *HBV* hepatitis B virus

^a^CI: confidence interval

Dependent variable: SGPT

Model summary: *F* = 11.92, *p* < 0.001

## Discussion

Despite the limitations of the present study, including the convenience sampling that limits the generalizability of the findings to all adults with disabilities in Taiwan, four key findings regarding liver health in disabled adult HBV or HCV carriers emerged from this study. First, there was a high prevalence of HCV infection in adults with disabilities, especially in older people and those with a low socioeconomic status. Second, few people are aware of viral infections, and very few receive antiviral treatment, which are effective and can successfully cure the infection. Third, most of the disabled participants with or without hepatitis reported some specific unhealthy behaviors. Fourth, high fasting blood glucose levels, overweight, and alcohol consumption were important and preventable risk factors, in addition to HBV/HCV infection and older age.

The prevalence rate of HBV in the present study (12.9 %) was lower than the nationwide prevalence (15–20 %), but higher than those of the adult population in different geographical areas, including sub-Saharan Africa and East Asia (5–10 %), the Middle East (2–5 %), and Western Europe and North America (1 %) [1–4]. Therefore, although the number of newly acquired HBV infections in Taiwan has declined substantially since the implementation of the national immunization program, the prevalence of chronic HBV and HCV infections remains high in some rural areas [3, 10, 12], including the western coastal areas (HBV, 18.7 %; HCV, 20.8 %) [10].

Based on the results of the present study (Table 2), possible reasons for higher rates of HCV infection in adults with disabilities living in rural areas (14.1 %) than the nationwide prevalence (2–3 %) include health disparity resulting from the limited resources and unqualified medical personnel using unsterilized materials [4], lack of education, poverty, illiteracy, and lack of information about virus-related hepatitis. Although there is currently no vaccine for HCV, evidence strongly suggests that some antiviral drugs are effective for eradicating the entire virus and reducing the incidences of hepatic cancer by 78 % and liver cirrhosis by 47 % [1–4]. Fortunately, antiviral treatments, such as interferon, lamivudine, adefovir, and ribavirin are covered by the Taiwan national health insurance [4]. Therefore, strategies to enhance the awareness of virus infection and motivate disabled residents to receive and complete the entire course of antiviral treatments are important issues for clinicians and primary health care providers.

The present study also revealed that many disabled participants have unhealthy behaviors, such as inadequate exercise and insufficient water, fruit, and vegetable intake. Moreover, 9.1 and 6.6 % of the participants reported current and previous alcohol consumption, respectively (Table 2). Although these habits were not directly correlated with health status, previous studies have reported that insufficient exercise, and inadequate water, fruit, and vegetable intake were significantly associated with obesity/overweight and HCC [20–23]. Furthermore, poor adherence to a Mediterranean diet was particularly detrimental for the risk of HCC in chronically infected HBV and/or HCV carriers [22, 23].

The modifiable factors significantly associated with elevated liver function included high fasting blood glucose levels, overweight, and alcohol drinking in the final regression model, in addition to HBV/HCV infection. Similarly, a previous study indicated that important lifestyle factors, such as alcohol consumption, diabetes, and obesity, also contribute to HCC [20], and increasing evidence is emerging that obesity is a major etiology for abnormal liver function among the young generation [24]. A meta-analysis of cohort studies reported a 17 and 90 % increased risk of HCC for overweight and obese people, respectively, compared with those of normal weight [25]. The association between obesity and non-alcoholic fatty liver disease could explain this increased risk [22–24]. In addition, HBV or HCV carriers in an Aboriginal population with a high incidence of alcohol consumption and smoking also had the most elevated levels of biomarkers of abnormal liver function [26].

Considering the limitations associated with physical, mental, or multiple disabilities among this vulnerable population, the inclusion of healthy lifestyle modifications for people with disabilities living in rural areas is an important strategy for primary health care providers. In addition, more convenient HBV vaccine booster programs could be provided for disabled young people whose HBV antibodies are no longer present following the 3 vaccination doses in infancy. Furthermore, access to antiviral treatments at 6–12-month periods for HCV carriers with disabilities is an important strategy for health care providers and district governments. Moreover, further studies are necessary to explore and initiate potential health promotion programs that encourage regular monitoring (e.g., every 3–6 months) for the early diagnosis of chronic liver disease, adoption of appropriate therapies, and avoiding overuse of alcohol and smoking for community-dwelling adults with disabilities.

### Limitations

This study had certain limitations. First, the participants were not randomly recruited and were from the same geographic area, Chiayi County, which limits the generalizability of these findings. Second, the descriptive study design does not allow for causality to be established, and possible confounders might not have been measured. Third, self-reporting often results in underestimation of unhealthy behaviors, such as the amount and type of alcohol consumed and extent of cigarette smoking. Finally, selection and recall bias need to be considered because of the various health conditions of the participants.

## Conclusions and implications

There was a high prevalence of HCV infection among adults with disabilities in the present study; few of the participants possessed an awareness of their liver health, and very few received early treatment or information on the prevention of liver cancer. Despite the presence of some unmodifiable risk factors, early detection of unhealthy behaviors and referral for further diagnosis of HBV/HCV carriers, increasing awareness of virus-related hepatitis and its consequences, providing accessible HBV vaccine booster programs and antiviral treatments for HCV carriers, and initiating health promotion programs are important health strategies for these minority adults.
